# Categorical and dimensional approaches to the developmental relationship between ADHD and irritability

**DOI:** 10.1111/jcpp.13818

**Published:** 2023-05-11

**Authors:** Rania Johns‐Mead, Nandita Vijayakumar, Melissa Mulraney, Glenn Melvin, George Youssef, Emma Sciberras, Vicki A. Anderson, Jan M. Nicholson, Daryl Efron, Philip Hazel, Tim J. Silk

**Affiliations:** ^1^ Centre for Social and Early Emotional Development (SEED) and School of Psychology Deakin University Geelong Vic. Australia; ^2^ Institute for Social Neuroscience ISN Psychology Ivanhoe Vic. Australia; ^3^ Murdoch Children's Research Institute Melbourne Vic. Australia; ^4^ The Royal Children's Hospital Melbourne Vic. Australia; ^5^ Department of Paediatrics University of Melbourne Melbourne Vic. Australia; ^6^ Judith Lumley Centre La Trobe University Melbourne Vic. Australia; ^7^ Sydney Medical School, Faculty of Medicine and Health The University of Sydney Sydney NSW Australia

**Keywords:** ADHD, irritability, latent change score, longitudinal

## Abstract

**Background:**

Attention deficit hyperactivity disorder (ADHD) and irritability commonly co‐occur, and follow similar developmental trajectories from childhood to adolescence. Understanding of the developmental relationship between these co‐occurrences is limited. This study provides a longitudinal assessment of how ADHD diagnostic status and symptom patterns predict change in irritability.

**Methods:**

A community sample of 337 participants (45.2% ADHD), recruited for the Childhood Attention Project, completed the Affective Reactivity Index (ARI) to measure irritability at baseline (mean age 10.5 years) and follow‐up after 18‐months. Latent change score models were used to assess how (a) baseline ADHD vs. control group status, (b) baseline symptom domain (inattention, hyperactivity–impulsivity) and (c) longitudinal change in ADHD symptom severity predicted change in irritability.

**Results:**

Irritability was significantly higher among the ADHD group than controls; however, change in irritability over time did not differ between groups. When assessed across the entire cohort, change in irritability was predicted by higher symptom count in the hyperactive–impulsive domain, but not the inattentive domain. Greater declines in ADHD symptoms over time significantly predicted greater declines in irritability. Baseline ADHD symptom severity was found to significantly predict change in irritability; however, baseline irritability did not significantly predict change in ADHD symptoms.

**Conclusions:**

ADHD symptoms—particularly hyperactive–impulsive symptoms—predict the degree and trajectory of irritability during childhood and adolescence, even when symptoms are below diagnostic thresholds. The use of longitudinal, dimensional and symptom domain‐specific measures provides additional insight into this relationship.

## Introduction

Attention deficit hyperactivity disorder (ADHD) and irritability are developmentally sensitive constructs, which contribute to functional impairment and mental health service referrals among children and adolescents (Stringaris, Vidal‐Ribas, Brotman, & Leibenluft, 2018). Irritability refers to an individual's tendency to anger quickly and/or easily, at a lower threshold of frustration or threat than is considered developmentally appropriate (Brotman, Kircanski, & Leibenluft, 2017). Overlap with related terms, including emotional impulsivity, emotion dysregulation and lability arises when they are used in relation to anger responses.

Although not core symptoms of ADHD, irritability, low frustration tolerance and mood lability are considered associated features (APA, 2022) and are more prevalent among children with ADHD than those without (Mayes et al., 2015). The nature and longitudinal stability of this association is not known. Previous studies have found that between 20% and 50% of children with ADHD experience elevated irritability and difficulties with emotion dysregulation (Ambrosini, Bennett, & Elia, 2013; Shaw, Stringaris, Nigg, & Leibenluft, 2014; Stringaris & Goodman, 2009). Mayes et al. (2015) found that irritable/angry mood and temper outbursts were experienced by 39% of children with ADHD‐Combined type (*N* = 570), and 12% of children with ADHD‐Inattentive type (*N* = 257), compared to 3% of neurotypical children (*N* = 186). Along with findings that hyperactive–impulsive symptom severity predicts aggression and externalising difficulties, even at levels below the diagnostic threshold (Connor & Ford, 2012), this suggests irritability in ADHD may be related to hyperactivity–impulsivity.

Irritability and impulsivity are thought to have similar neurological underpinnings (Chaarani et al., 2020) and overlap in their association with executive and regulatory functions such as cognitive control (Leibenluft, 2017). Dysregulation of both positive and negative emotions has been linked to impulsive risk taking (Weiss, Sullivan, & Tull, 2015), while dysregulation of negative emotions (i.e., irritability) may be associated with the disruptive behaviours observed in hyperactive–impulsive ADHD presentations, oppositional defiant disorder (ODD) and conduct disorder (Martel, 2009). It has been suggested that impulsive behaviour in ADHD may in part be driven by the influence of unregulated emotional responses on goal‐directed behaviour (Faraone et al., 2019; Martel, 2009), or that emotional symptoms including irritability represent a core symptom or characterise a subvariant of ADHD (Barkley, 2015; Karalunas, Gustafsson, Fair, Musser, & Nigg, 2019; Shaw et al., 2014). Others argue that irritability is independent of ADHD symptomology and linked only through association with comorbidities such as ODD and depression (Factor, Reyes, & Rosen, 2014).

ADHD symptoms and irritability have both been found to decline over development. Specifically, hyperactive–impulsive symptoms typically decrease, while inattentiveness shows little or no reduction (Biederman, Mick, & Faraone, 2000; Larsson, Lichtenstein, & Larsson, 2006). In neurotypical populations, irritability tends to decline from early childhood to late adolescence (Brotman, Kircanski, Stringaris, Pine, & Leibenluft, 2017; Copeland, Brotman, & Costello, 2015; Leibenluft, Cohen, Gorrindo, Brook, & Pine, 2006). Despite their frequent co‐occurrence and apparent developmental similarities, it remains unclear how irritability changes over time among children with ADHD, whether this differs from typically developing children or how it may vary based on symptom domain (hyperactive–impulsive/inattentive), severity and course.

Research in this area has faced a number of methodological challenges, which the current study aims to address. First, the heterogeneity of the ADHD population is not captured by categorical analysis of groups based on diagnostic classification. There is growing support for the inclusion of dimensional assessment in research, endorsed by the National Institute of Mental Health Research Domain Criteria (RDoC) framework (Musser & Raiker, 2019). Such an approach would enable consideration of differing severity and symptom patterns in ADHD, and their implications for changes in irritability across development (Heidbreder, 2015). Second, measurement of irritability is inconsistent, and often undertaken by extracting and combining items from existing scales and diagnostic interview protocols (Vidal‐Ribas, Brotman, Valdivieso, Leibenluft, & Stringaris, 2016). This may be due to a focus on irritability that meets a threshold of clinical interest (e.g. disruptive mood dysregulation disorder or ODD) or opportunistic utilisation of irritability‐related items included in other measures. While resourceful Vidal‐Ribas et al. (2016), note this may result in problems with reliability and validity and suggest the use of specifically designed measures such as the Affective Reactivity Index (ARI). Finally, most literature on irritability in ADHD is cross‐sectional. The few longitudinal studies focus on change in diagnostic status or identification of distinct profiles and tend to use categorical classifications. For example, Riglin et al. (2019) found that chronically high irritability is associated with ADHD. However, they focus on the relationship of discrete irritability profiles with diagnostic categories, and use an ad hoc measure of irritability as described by Vidal‐Ribas et al. (2016). No studies have been identified which examine the relationship between change in ADHD symptom severity and change in irritability over time.

This study aimed to investigate longitudinal change in irritability from late childhood to early adolescence, and determine whether this differs based on (a) childhood history of diagnosed ADHD (ADHD/control), (b) symptom count in each domain (inattentive/hyperactive–impulsive) and (c) change in ADHD symptom severity. First, it was predicted that irritability would be higher, and decline more slowly among the ADHD group than among controls. Second, that higher symptom counts—particularly for hyperactivity–impulsivity—would predict higher irritability with less decline over time. Lastly, that severity of ADHD symptoms would positively correlate with concurrent irritability, that greater severity of ADHD symptoms at baseline would predict smaller declines in irritability while greater irritability at baseline would predict smaller declines in ADHD symptoms and that change in ADHD symptoms would be positively associated with change in irritability.

## Method

Data were collected as a part of the Children's Attention Project, a longitudinal project studying a community‐based sample, recruited across 43 primary schools in Melbourne, Australia. For full details, see Sciberras et al. (2013). Commencing in grade 1 (mean age 7.3 years), participants underwent up to five waves of data collection at approximately 18‐month intervals (see Table S1). The Diagnostic Interview Schedule for Children‐IV (DISC‐IV; Shaffer, Fisher, Lucas, Dulcan, & Schwab‐Stone, 2000) was used to confirm ADHD diagnostic status at waves 1 and 3. The current study focused on waves 3 and 4, in which irritability was assessed and will subsequently refer to these time points as baseline (mean age 10.5 years) and 18‐month follow‐up.

Approval for this project was granted by The Royal Children's Hospital (#31056 and #34071) and the Deakin University Human Research Ethics Committee (#2016‐394).

### Participants

**Figure 1:**
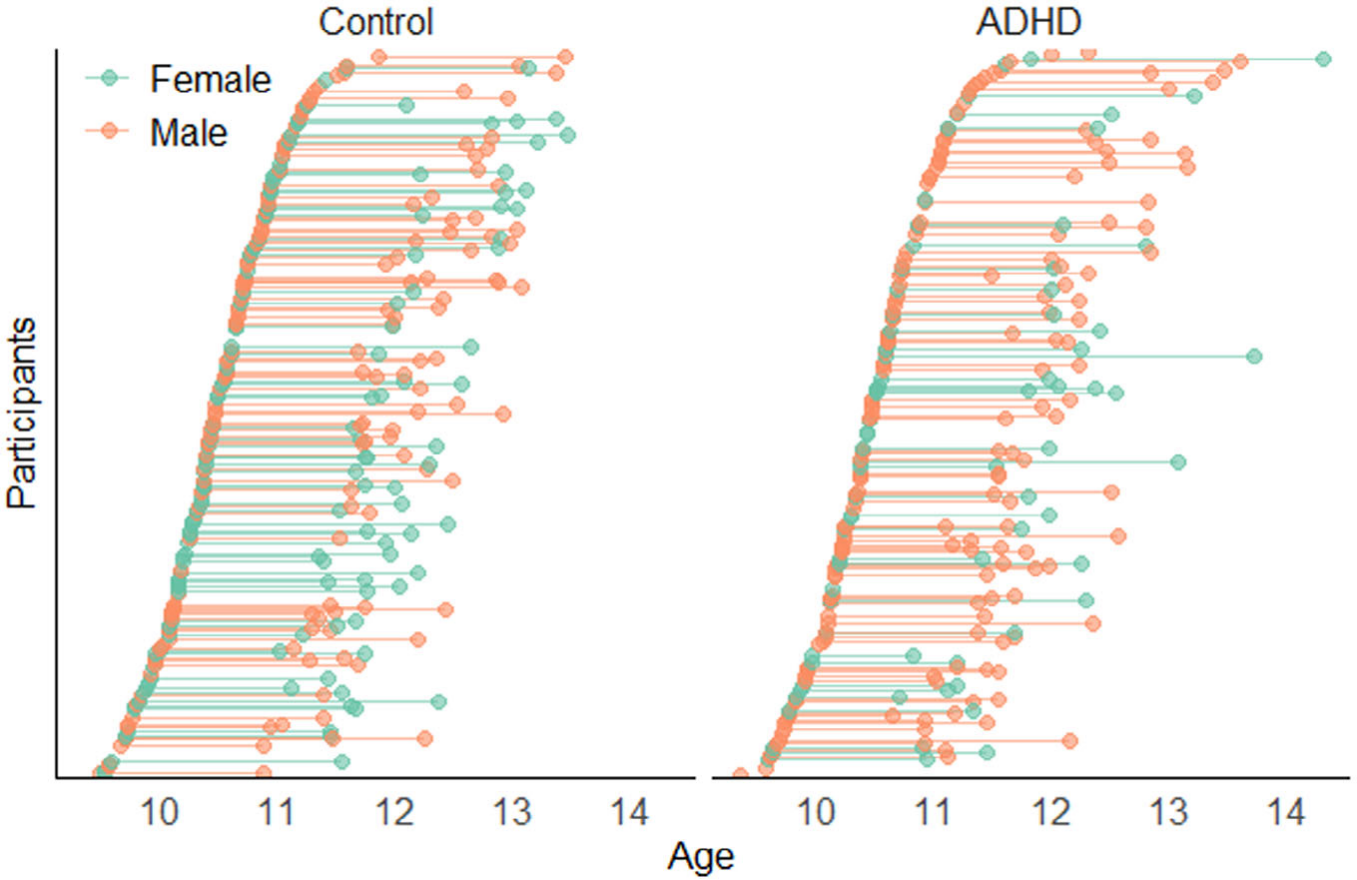
Participant age at baseline and follow up, by ADHD status and sex [Color figure can be viewed at wileyonlinelibrary.com]

Data were included for 337 children who completed the ARI at baseline. In all, 42 cases with missing data for ARI at baseline were removed. The sample had a mean age of 10.49 (*SD* 0.50) years at baseline, with a mean interval of 1.57 (*SD* 0.37) years to follow‐up (see Figure 1). Of this sample, 208 (61.71%) were males, and 152 (45.24%) met ADHD criteria on the DISC‐IV at one or more time points (see Table S2). Of those with a childhood history of ADHD, 44 (28.95%) were prescribed medication for their behavioural difficulties, as were 6 (3.26%) controls. See Table 1 for sample characteristics. Descriptive statistics for longitudinal variables are presented in Table S3.

**Table 1:** Sample characteristics at baseline (mean age 10.5 years)

	ADHD (*n* = 152)		Control (*n* = 184)	
	*N*	%	*N*	%
Male	108	71.05	100	54.35
Internalising disorder	33	21.71	15	8.15
Externalising disorder	70	46.05	18	9.78
Medication	44	28.95	6	3.26
Medication (multiple)	22	14.47	1	0.54

Percentage is of total cases in each group (ADHD, control) including missing data. Missing data: ADHD status = 1; sex = 0; internalising disorder = 16; externalising disorder = 16; medication = 1; medication (multiple) = 2. Breakdown by medication type presented in Table S4. ADHD, attention deficit hyperactivity disorder.

### Measures

#### ARI: irritability

The ARI is a dimensional measure of irritability in the last 6 months (Stringaris et al., 2012). This study used the parent‐report ARI, which has been validated for use among children and adolescents, with internal consistency in the general population ranging from 0.80 in an Australian sample (Mulraney, Melvin, & Tonge, 2014) to 0.92 in a US sample (Stringaris et al., 2012). The ARI comprises six items relating to children's feelings or behaviours (e.g. ‘Lose temper easily’) and one relating to impairment (‘Overall, irritability causes them problems’). Responses comprise a three‐point Likert scale ranging from 0 (not true) to 2 (certainly true). The total irritability score was derived by summing the first 6 items (range: 0–12), whereby higher scores indicate more severe irritability.

#### DISC‐IV: ADHD status, inattentiveness, hyperactivity–impulsivity and other mental disorders

The DISC‐IV is a structured diagnostic interview, designed to assess DSM‐IV disorders in children and adolescents, and validated for use in clinical and community samples (Shaffer et al., 2000). The DISC‐IV was administered at recruitment and repeated at baseline (mean age 10.5 years). Participants who met ADHD diagnostic criteria at either time were assigned to the ADHD group. Those who did not meet criteria were defined as controls. Also collected at baseline were symptom counts for the inattentive and hyperactivity–impulsivity domains. Other mental disorders were assessed at baseline and included internalising (separation anxiety disorder, social phobia, generalised anxiety disorder, post‐traumatic stress disorder, obsessive–compulsive disorder, hypomania/manic episode) and externalising (ODD or conduct disorder) disorders. DISC‐IV data were operationalised as three dichotomous variables (ADHD group, internalising disorder and externalising disorder) and two continuous variables (domain‐specific symptom counts).

#### C‐3AI: dimensional measure of ADHD symptoms

The C‐3AI is a standalone index developed as a part of the Conners 3rd Edition, indicating the likelihood of an individual meeting DSM diagnostic criteria for ADHD. This study used the parent‐report version at baseline and follow‐up, which assess ADHD symptoms among 6‐ to 16‐year‐olds. It comprises 10 items, scored on a 4‐point scale. Higher scores indicate more severe symptoms.

### Analyses

Statistical analyses were conducted using Mplus version 7 (Muthén & Muthén, 1998–2012, Los Angeles, USA). Figures were created using RStudio (RStudio Team, 2020, Boston, USA). Data were screened for out‐of‐range values. Missing data ranged from 0% to 4.7% for baseline and single time point measures, and from 22.6% to 23.1% at follow‐up. Investigation of missing data showed attrition varied only by age, such that those without follow‐up data had a median age of approximately 2.4 months older than those with. Accordingly, full information maximum likelihood was used, and a robust maximum likelihood estimator (with a significance threshold of *p* < .05) employed, for all the analyses. All the analyses used the same sample, were adjusted for age, sex, medication status, internalising disorders, externalising disorders and baseline scores for longitudinal measures.

Research questions were addressed through three analyses conducted using latent change score (LCS) models, a class of structural equation model developed for longitudinal data which may interact over time, which can be used with as few as two time points (Kievit et al., 2018). This approach uses observed variables to test hypotheses regarding latent change variables, modelling change at the construct level. Univariate LCS models can be applied similarly to a paired samples *t*‐test but have the advantage of enabling estimation of variance within the change factor, and capturing the extent to which change is dependent on baseline levels (Kievit et al., 2018). Multivariate LCS models can be applied to multiple longitudinal variables with the same number of repeated measures taken at the same time points, to assess covariance at baseline, cross‐domain coupling (the extent to which change in one domain is predicted by baseline levels of the other) and correlated change (the extent to which change in one domain predicts change in the other).

Initially, univariate change in longitudinal variables of interest (ARI and C‐3AI) was examined using observed scores at baseline and follow‐up to model the latent change in each variable over time. These univariate models identified the direction and significance of latent change in irritability and ADHD symptoms, and the degree to which latent change is predicted by baseline levels, informing how relationships of other variables to latent change were interpreted in subsequent analyses.

Aim 1 was examined using a univariate LCS model of ARI, separately by ADHD group status, to examine differences in ARI between ADHD and control groups. The Wald test was used to compare between‐group differences across (a) baseline ARI scores, (b) latent change in ARI and (c) relationship between baseline ARI and latent change in ARI. For robustness testing, a supplementary analysis was also conducted in which participants taking medication at either time point were removed (see Supporting Information). Aim 2 was examined using a univariate LCS model of ARI, with inattention and hyperactivity symptom counts at baseline included simultaneously as predictor variables of interest. This analysis tested whether these variables significantly predict latent change in ARI and examined their relationship to baseline ARI. Aim 3 was examined using a bivariate latent change model to assess baseline covariance, cross‐domain coupling and simultaneous latent change of C‐3AI ADHD symptoms and irritability.

## Results

### Univariate models of longitudinal variables

Estimated univariate models of ARI and C‐3AI identified a statistically non‐significant negative mean latent change in both variables (i.e. improving; see Table 2). They also identified a significant relationship between baseline ARI and latent change in ARI (−.309, *SE* = .054, *p* < .001), and between baseline C‐3AI and latent change in C‐3AI (−.403, *SE* = .048, *p* < .001), such that higher levels at baseline were associated with less change (i.e. smaller decreases) over time. Thus, subsequently described LCS models account for these relationships by covarying for baseline variables.

**Table 2:** Univariate descriptive statistics

Variable	Baseline			Follow‐up			Latent change		
	*M*	*SD*	*N*	*M*	*SD*	*N*	Δ	*SE*	*p*
ARI	3.282	3.253	337	3.120	3.407	259	−0.229	0.146	0.118
C‐3AI	5.395	6.399	337	5.042	6.095	261	−0.456	0.235	0.052

ARI, Affective Reactivity Index; C‐3AI, Conners‐3 ADHD Index; *M*, mean; *N*, number of subjects; *SD*, standard deviation; *SE*, standard error; Δ, estimated latent change.

### Group comparison of irritability

**Figure 2:**
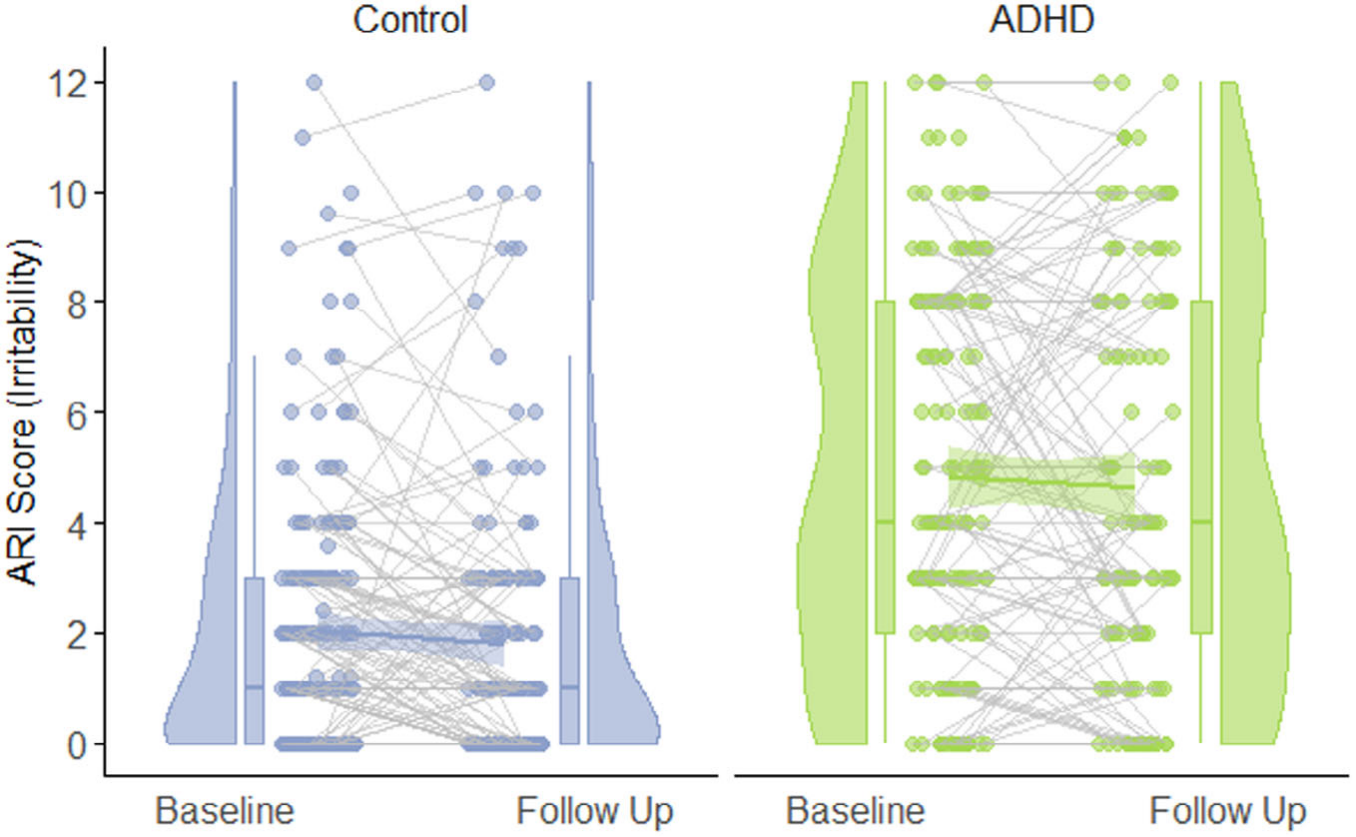
Change and variance in irritability by attention deficit hyperactivity disorder (ADHD) status [Color figure can be viewed at wileyonlinelibrary.com]

Baseline ARI was significantly higher among those with ADHD (*M* = 3.505, *SE* = .295) than those without (*M* = 2.380, *SE* = .246), χ^2^(1) = 12.306, *p* < .001. However, latent change in ARI was not significant within either group (see Table 3), and there was no significant group difference in magnitude of change, χ^2^(1) = 0.849, *p* = .357, indicating that, when baseline differences are accounted for, the magnitude of change in irritability is the same across groups independent of starting point. The relationship between baseline ARI and latent change in ARI did not differ between groups, χ^2^(1) = 0.432, *p* = .511. Figure 2 displays between‐group differences and within‐group variance in irritability over time. Within the ADHD group, covariates sex (*p* = .037) and medication (*p* = .001) significantly predicted change in ARI, such that greater declines in ARI were more likely among females, and participants taking medication. No covariates were significant within the control group.

**Table 3:** Latent change in ARI by ADHD status

Group	Δ	*SE*	*p*
Control	−1.800	3.342	.590
ADHD	−6.963	4.500	.122

Standardised results presented in Table S5. ADHD, attention deficit hyperactivity disorder; ARI, Affective Reactivity Index; *SE*, standard error; Δ, estimated latent change.

Results for supplementary analysis of unmedicated participants were consistent with whole sample analyses and are presented in the Tables S6 and S7.

### Irritability by symptom domain

LCS analysis of ARI with baseline inattention and hyperactivity–impulsivity found that number of hyperactive symptoms significantly predicted latent change in ARI. Higher baseline hyperactivity predicted a stronger magnitude of reduction in ARI (see Table 4). However, baseline inattentiveness did not significantly predict latent change in ARI. Neither symptom domain was significantly correlated with baseline ARI. Of the covariates, sex (*p* = .031), internalising disorder (*p* = .044) and medication (*p* = .017) accounted for a significant proportion of variance in change in ARI, such that greater declines in ARI were more likely among females, participants taking medication and participants with an internalising disorder at baseline. Greater baseline ARI scores were associated with externalising disorders (*p* < .001) and taking medication (*p* = .014).

**Table 4:** Baseline ARI and latent change in ARI by symptom domain

	*B*	*SE*	*p*
Baseline			
Hyperactive–impulsive	.145	0.075	.053
Inattentive	.098	0.053	.066
Latent change			
Hyperactive–impulsive	.183	0.071	**.010**
Inattentive	−.030	0.051	.553

Standardised results presented in Table S8. ARI, Affective Reactivity Index; *B*, unstandardised coefficient; *SE*, standard error.

Bold indicates *p* < 0.05.

### Irritability by symptom severity

**Figure 3:**
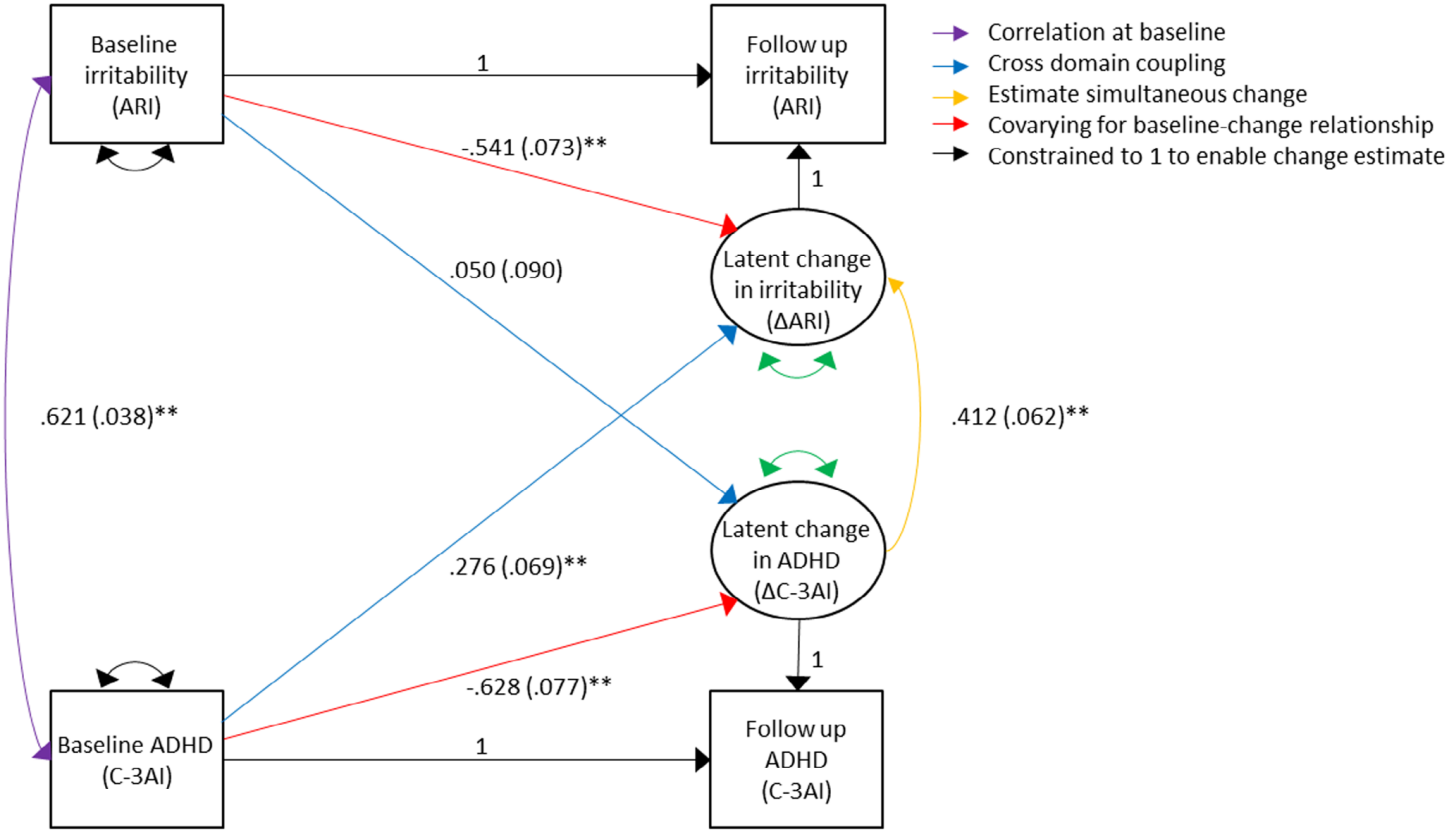
Bivariate LCS model of change in irritability and change in ADHD symptoms. Standardised regression coefficients are presented as β(*SE*)^*p*^. **p < .001. Unstandardised results presented in Figure S1. ADHD, attention deficit hyperactivity disorder [Color figure can be viewed at wileyonlinelibrary.com]

Analysis of ARI and C‐3AI identified a significant relationship at baseline (see Figure 3) such that higher C‐3AI scores were associated with greater ARI scores. The cross‐coupling relationships show that baseline C‐3AI significantly predicted latent change in ARI such that higher severity predicts greater reductions in irritability, while baseline ARI did not significantly predict change in C‐3AI. Latent change in C‐3AI was also found to predict simultaneous latent change in ARI. As univariate analyses revealed mean latent change to be negative for both variables, the positive coefficient of their relationship indicates that a greater magnitude of change in ADHD symptoms was associated with a greater magnitude of change in irritability in the same direction. A larger decrease in ADHD symptoms predicted a correspondingly larger decrease in irritability.

Of the covariates, sex (*p* = .014) and medication (*p* = .038) significantly predicted change in ARI, such that greater declines in ARI were more likely among females, and those taking medication. Greater declines in C‐3AI were more likely among older participants (*p* = .041), those with an externalising disorder (*p* = .008) and those taking medication (*p* = .008). At baseline, ARI and C‐3AI were both significantly higher among those with an internalising disorder (*p* < .001), an externalising disorder (*p* < .001) and those taking medication (*p* < .001).

## Discussion

This study aimed to examine how irritability changes across late childhood and early adolescence in relation to childhood history of diagnosed ADHD, symptom domain and changes in symptom severity over time. Results found that irritability was higher among those with ADHD than among controls, but followed a similar trajectory over time. Moreover, greater baseline hyperactivity–impulsivity predicted declining irritability over time. Finally, declining ADHD symptoms were found to predict corresponding declines in irritability, demonstrating a longitudinal relationship between these factors when measured dimensionally.

The finding that irritability was higher among those with ADHD than controls was in keeping with predictions and with previous findings (Mayes et al., 2015; Shaw et al., 2014). Counter to predictions, no group differences were found in magnitude or direction of change, suggesting that differences identified at baseline were stable. While ADHD has been associated with irritability cross‐sectionally, this finding provides initial evidence that differences in irritability between ADHD and typically developing populations represent parallel trajectories during late childhood and early adolescence.

Although analysis of the relationship of irritability to ADHD symptom counts in each domain revealed a relationship between hyperactivity–impulsivity and latent change in irritability, neither symptom domain significantly predicted baseline irritability, despite an observed trend in the expected direction. This is in contrast to our predictions and previous studies linking hyperactivity to irritability and impulsive aggression (Connor & Ford, 2012; Mayes et al., 2015). Moreover, hyperactivity–impulsivity predicted greater reductions, rather than increasing or persisting irritability. While this suggests a dimensional relationship between level of hyperactivity–impulsivity and change in irritability, this unexpected finding warrants further discussion in the context of other results.

Finally, as predicted, the results identified positive relationships between ADHD severity and irritability concurrently at baseline, over time in the form of cross‐domain coupling and with change in ADHD severity predicting synchronous change in irritability. Baseline irritability, however, did not significantly predict change in ADHD symptoms. These results are consistent with previous findings that ADHD and irritability are commonly comorbid (Ambrosini et al., 2013; Mayes et al., 2015; Mulraney, Zendarski, & Coghill, 2021; Shaw et al., 2014; Stringaris & Goodman, 2009), and show similar declines over the course of development (Biederman et al., 2000; Brotman, Kircanski, Stringaris, et al., 2017; Copeland et al., 2015; Larsson et al., 2006; Simon, Czobor, Bálint, Mészáros, & Bitter, 2009). This study demonstrates that the observed patterns of similarity in the existing literature represent a significant relationship in which severity of, and changes in, ADHD symptoms predict change in irritability such that declining ADHD symptom severity predicts simultaneous declines in irritability.

### Implications

Our findings describe the typical patterns of irritability in late childhood and early adolescence, as they relate to ADHD and ADHD symptoms. As irritability is a transdiagnostic symptom, understanding longitudinal patterns is a necessary step towards differentiating between irritability as a feature of ADHD, and irritability as an indicator of comorbid psychopathology. This study supports the status of irritability as an associated feature of ADHD (APA, 2022) rather than being attributable to co‐occurring disorders, as suggested by Factor et al. (2014). Significant relationships with ADHD variables remained in each analysis, while covarying for internalising and externalising disorders—even where significant relationships of these variables to irritability were also noted.

This study also indicates the relationship between irritability and ADHD symptoms extends beyond diagnostic thresholds. While categorical ADHD status did not predict change in irritability independently of baseline levels, dimensional measurement of hyperactive–impulsive symptoms, and ADHD severity did. This demonstrates the value of dimensional measurement and provides further evidence that ADHD symptoms—particularly hyperactive–inattentive symptoms—may influence the degree and trajectory of irritability, even for individuals without an ADHD diagnosis (Connor & Ford, 2012). In light of findings that subthreshold ADHD symptoms are associated with functional impairment in children (Efron et al., 2020; Kirova et al., 2019; Zendarski et al., 2020), and increased rates of self‐harm, suicidal ideation and intent in adolescents (Mulraney et al., 2021), this study adds to the growing body of evidence that ADHD‐like symptoms are an important clinical consideration, even when full diagnostic criteria are not met.

These results build upon prior research suggesting a specific relationship between irritability and hyperactive–impulsive symptoms (Connor & Ford, 2012; Mayes et al., 2015). Although the finding that greater baseline levels of hyperactivity–impulsivity predicted reductions in irritability was unexpected, existing literature provides a possible explanation. Previous studies show that hyperactivity–impulsivity typically decreases with age, while inattentiveness remains stable (Biederman et al., 2000; Larsson et al., 2006). It may be that the decrease in irritability predicted by higher baseline hyperactivity–impulsivity was accompanied by a concurrent decrease in hyperactivity–impulsivity (which was not measured longitudinally in this data set)—that is, that declining irritability was identified among individuals with initially high, but subsequently decreasing hyperactivity–impulsivity. Whilst beyond the current study's scope, this possibility is consistent with our findings that declining ADHD symptoms predict declining irritability. Although the latter result did not differentiate between symptom domains, a reasonable inference could be made based on knowledge of typical symptom trajectories in this period (Biederman et al., 2000; Lahey & Willcutt, 2010; Larsson et al., 2006) that decreasing ADHD severity is likely to reflect changes in hyperactive–impulsive symptoms rather than inattention, which is typically stable. This supposition is also consistent with conceptualisations of irritability in ADHD as a form of emotional impulsivity (Barkley, 2010; Faraone et al., 2019), arising from self‐regulation difficulties equivalent to those underlying impulsivity. Furthermore, it is consistent with treatment recommendations that irritability in the context of ADHD may be addressed in the course of treating ADHD symptoms via both pharmacological and psychosocial interventions (Daley et al., 2018; Fernández de la Cruz et al., 2015).

### Strengths, limitations and future directions

The current study benefits from a number of strengths. It used a large, longitudinal, community‐based sample, increasing the power and generalisability of results, and allowing for a developmental perspective on ADHD and irritability. It also operationalised irritability dimensionally to better capture change, in contrast to less sensitive categorical measures. A range of ADHD variables were used to increase the theoretical and clinical utility of findings. Using symptom domain and dimensional approaches to account for heterogeneity (Heidbreder, 2015; Musser & Raiker, 2019), this study identified relationships that the categorical‐diagnostic approach was not sensitive to. This further highlights the importance of dimensional approaches, and is in keeping with the RDoC principles. Finally, this study used LCS modelling, a novel statistical approach in this research area, to effectively utilise the longitudinal data. This allowed modelling of concurrent change, influence of baseline differences and variance in change score estimates. A further advantage is the ability comment on causal direction through interpreting cross‐domain coupling in bivariate models—a challenge for developmental research. In this case, LCS analysis enabled the inference that irritability is influenced by ADHD symptoms rather than the reverse—a finding beyond the scope of traditional regression models.

Findings must also be considered in light of limitations. As discussed, domain‐specific symptom data were not collected longitudinally; thus, the hypothesis that declines in irritability may be explained by concurrent decreases in hyperactivity–impulsivity could not be tested. Future studies should include longitudinal, domain‐specific measures of ADHD symptoms to interrogate this explanation. Moreover, the distributions of both the ARI and the C‐3AI are positively skewed and may be less sensitive to change at the lower end of the spectrum if a floor effect occurs. Additionally, the C‐3AI is designed as a brief indicator of overall ADHD symptoms, rather than a comprehensive measure of total symptoms present. While these tools have effectively demonstrated the value of dimensional investigation using a novel statistical approach, future research would benefit from applying similar methods to more comprehensive measures, such as long version of the Conners 3^rd^ Edition or the Strengths and Weaknesses of Attention‐Deficit/Hyperactivity‐symptoms and Normal‐behaviors (SWAN), which evaluates normal and abnormal variance in ADHD symptoms. This would enable more in‐depth characterisation of change in ADHD symptoms over time.

Furthermore, irritability is known to change from mid‐childhood to late adolescence, and some literature suggests it may have a curvilinear trajectory (Brotman, Kircanski, Stringaris, et al., 2017; Copeland et al., 2015; Leibenluft et al., 2006). Thus, future research should examine a wider age range, with multiple time points to capture change. While this study covaried for medication use, it was beyond its scope to investigate the impact of medical or psychological interventions for irritability in ADHD, nor does the current study comment on the underlying neurodevelopmental mechanisms of the relationship between ADHD and irritability. These, too, are important areas for future research.

## Conclusion

The current findings support the hypothesis that irritability is associated with ADHD and not solely attributable to comorbid disorders. It suggests that this relationship is not limited to cases above the diagnostic threshold, and that ADHD symptoms appear to influence changes in irritability, not the other way around. Finally, irritability in ADHD may be specifically related to hyperactive–impulsive symptoms, in keeping with conceptualisations of irritability in ADHD as a form of emotional impulsivity, rather than representing mood disturbance. Continued investigation is necessary to further understand the developmental relationship between irritability and ADHD and its implications for diagnosis and intervention.

## Supporting information


**Table S1.** Data collected at each wave by variables of interest.
**Table S2.** Diagnostic threshold for ADHD symptoms on the DISC‐IV at recruitment and baseline.
**Table S3.** Descriptive statistics for longitudinal variables.
**Table S4.** Supplement to Table 1.
**Table S5.** Supplement to Table 3.
**Table S6.** Latent change in ARI by ADHD status in unmedicated participants.
**Table S7.** Standardised results for latent change in ARI by ADHD status in unmedicated participants.
**Table S8.** Supplement to Table 4.
**Figure S1.** Supplement to Figure 3.
