# Supplementary material for: Categorical and dimensional approaches to the developmental relationship between ADHD and irritability
Source: J Child Psychol Psychiatry. 2023 May 11;64(10):1422–31. doi: 10.1111/jcpp.13818 (PMC10952727; doi:10.1111/jcpp.13818)
Supplement: Supplementary file 1 — Table S1. Data collected at each wave by variables of interest. Table S2. Diagnostic threshold for ADHD symptoms on the DISC‐IV at recruitment and baseline. Table S3. Descriptive statistics for longitudinal variables. Table S4. Supplement to Table 1. Table S5. Supplement to Table 3. Table S6. Latent change in ARI by ADHD status in unmedicated participants. Table S7. Standardised results for latent change in ARI by ADHD status in unmedicated participants. Table S8. Supplement to Table 4. Figure S1. Supplement to Figure 3. [file JCPP-64-1422-s001.docx]

The Developmental Relationship Between ADHD and Irritability:

Supporting Information

**Method**

**Table S1.**

*Data collected at Each Wave by Variables of Interest*

|  | Wave 1 | Wave 2 | Wave 3 | Wave 4 | Wave 5 |
| --- | --- | --- | --- | --- | --- |
| DISC-IV | X |  | X |  | X |
| ARI |  |  | X | X |  |
| C-3AI | X | X | X | X | X |

*Note.* DISC-IV = Diagnostic Interview Schedule for Children; ARI = Affective Reactivity Index.; C-3AI = Conners 3 ADHD Index.

## Participants

**Table S2.**

*Diagnostic Threshold for ADHD Symptoms on the DISC-IV at Recruitment and Baseline*

|  | Baseline | | |
| --- | --- | --- | --- |
| Recruitment | Above Threshold | Below Threshold | Unknown |
| Above Threshold | 81^a^ | 46^a^ | 6^a^ |
| Below Threshold | 20^a^ | 177^b^ | 4^b^ |
| Unknown | 1^c^ | 1^c^ | 1^c^ |

*Note.* DISC-IV = Diagnostic Interview Schedule for Children.

^a^ Childhood history of ADHD (ADHD group)

^b^ No childhood history of ADHD (control group)

^c^ Unknown history (excluded from groups analysis)

**Table S3.**

*Descriptive Statistics for Longitudinal Variables*

|  | N | Mean | SD | Skewness | Kurtosis |
| --- | --- | --- | --- | --- | --- |
| Baseline |  |  |  |  |  |
| ARI | 337 | 3.282 | 3.253 | .884 | -.259 |
| C-3AI | 337 | 5.395 | 6.399 | .926 | -.530 |
| Follow Up |  |  |  |  |  |
| ARI | 259 | 3.120 | 3.407 | 1.022 | -.077 |
| C-3AI | 261 | 5.042 | 6.095 | 1.084 | -.088 |
| Latent Change |  |  |  |  |  |
| ARI | 337 | -.206 | 2.161 | .242 | 2.644 |
| C-3AI | 337 | -.425 | 3.542 | -.603 | 3.345 |

*Note.* DISC-IV = Diagnostic Interview Schedule for Children; ARI = Affective Reactivity Index; C-3AI = Conners 3 ADHD Index.

**Table S4.**

*Supplement to Table 1. Sample Characteristics at Baseline (Mean Age 10.5 Years) with Breakdown by Medication Type*

|  | ADHD (152) | | Control (184) | |
| --- | --- | --- | --- | --- |
|  | N | % | N | % |
| Male | 108 | 71.05 | 100 | 54.35 |
| Internalising disorder | 33 | 21.71 | 15 | 8.15 |
| Externalising disorder | 70 | 46.05 | 18 | 9.78 |
| Medication | 44 | 28.95 | 6 | 3.26 |
| Ritalin 10mg | 12 | 7.89 | 1 | 0.54 |
| Ritalin LA | 5 | 3.29 | 1 | 0 |
| Concerta | 14 | 9.21 | 0 | 0 |
| Dexamphetamine | 1 | 0.66 | 0 | 0 |
| Strattera | 2 | 1.32 | 1 | 0.54 |
| Catapres | 6 | 3.95 | 1 | 0.54 |
| Risperidone | 7 | 4.61 | 0 | 0 |
| Lovan | 5 | 3.29 | 1 | 0.54 |
| Vyvanse | 6 | 3.95 | 0 | 0 |
| Melatonin | 17 | 11.18 | 1 | 0.54 |
| Other medication | 4 | 2.63 | 0 | 0 |
| Medication (multiple) | 22 | 14.47 | 1 | 0.54 |

*Note:* Percentage is of total cases in each group (ADHD, control) including missing data. Missing data: ADHD status = 1; sex = 0; internalising disorder = 16; externalising disorder = 16; medication = 1; medication (multiple) = 2.

**Results**

## Group Comparison of Irritability

**Table S5.**

*Supplement to Table 3. Standardised Results for Latent Change in ARI by ADHD Status*

|  | Δ | SE | *p* |
| --- | --- | --- | --- |
| Control group | -.877 | 1.584 | .580 |
| ADHD group | -2.505 | 1.587 | .114 |

*Note.* ARI = Affective Reactivity Index; Δ = standardised estimated of latent change; SE = standard error.

***Results for Sensitivity Analysis of Unmedicated Children.*** Baseline ARI was significantly higher among those with ADHD (M = 3.230, SE = .295) than those without (M = 2.067, SE = .195), χ^2^(1) = 12.966, *p* < 0.001. However, latent change in ARI was not significant within either group (see Supplementary Table 3), and there was no significant group difference in magnitude of change, χ^2^(1) = 2.023, *p* = 0.155, indicating that, when baseline differences are accounted for, the magnitude of change in irritability is the same across groups independent of starting point. The relationship between baseline ARI and latent change in ARI did not differ between groups, χ^2^(1) = 0.077, *p* = 0.782.

**Table S6.**

*Latent Change in ARI by ADHD Status in Unmedicated Participants*

|  | Δ | SE | *p* |
| --- | --- | --- | --- |
| Control | -1.321 | 3.382 | .696 |
| ADHD | -10.195 | 5.244 | .052 |

*Note.* N = 285; ARI = Affective Reactivity Index; Δ = estimated latent change; SE = standard error. Standardised results presented in supplementary materials.

**Table S7.**

*Standardised Results for Latent Change in ARI by ADHD Status in Unmedicated Participants*

|  | Δ | SE | *p* |
| --- | --- | --- | --- |
| Control group | -0.646 | 1.621 | .690 |
| ADHD group | -3.656 | 1.834 | .046 |

*Note.* N = 285; ARI = Affective Reactivity Index; Δ = standardised estimated of latent change; SE = standard error.

## Irritability by Symptom Domain

**Table S8.**

*Supplement to Table 4. Standardised Results for Baseline ARI and Latent Change in ARI by Symptom Domain*

|  | β | SE | *p* |
| --- | --- | --- | --- |
| Baseline |  |  |  |
| Hyperactive-impulsive | .118 | .061 | .052 |
| Inattentive | .098 | .053 | .066 |
| Latent Change |  |  |  |
| Hyperactive-impulsive | .202 | .078 | .**010** |
| Inattentive | -.041 | .069 | .552 |

*Note.* β = standardised coefficient; SE = standard error.

## Irritability by Symptom Severity

**Figure S1*.***

*Supplement to Figure 3. Unstandardised Bivariate LCS model of change in irritability and change in ADHD symptoms*


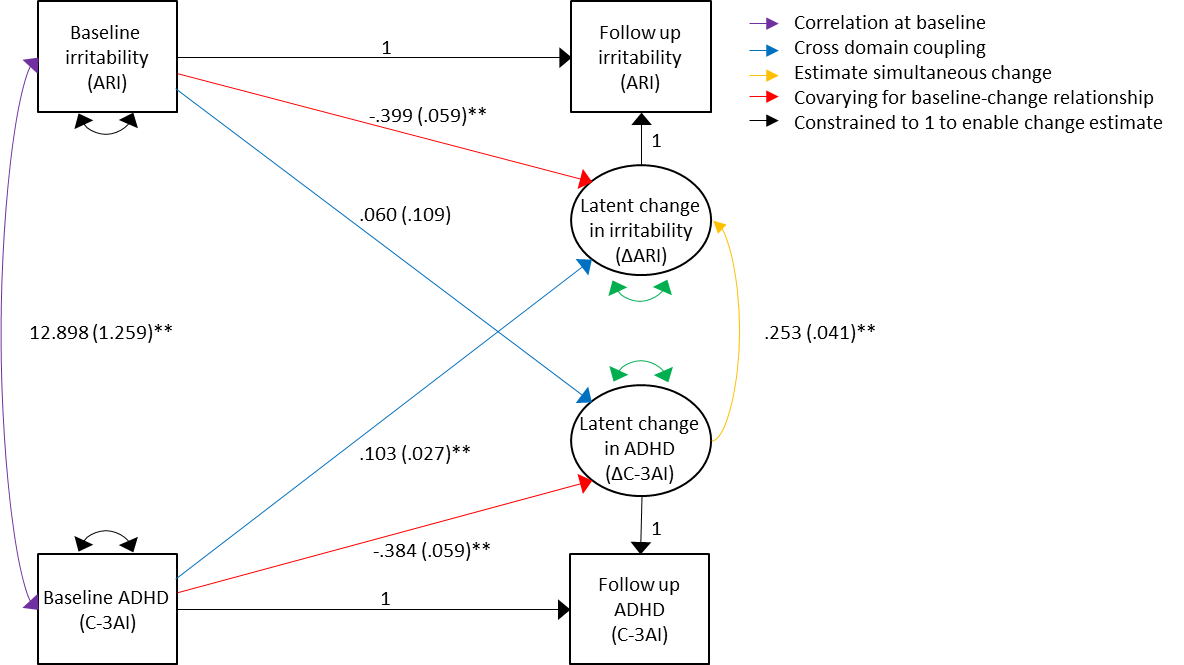


*Note.* Unstandardised regression coefficients are presented as B(SE)*^p^*. Every regression is covariate-adjusted for age, sex, medication status, and comorbid internalising and externalising disorders at baseline.

** *p* < .001
